# Test–retest reliability of brain morphology estimates

**DOI:** 10.1007/s40708-016-0060-4

**Published:** 2017-01-05

**Authors:** Christopher R. Madan, Elizabeth A. Kensinger

**Affiliations:** Department of Psychology, Boston College, McGuinn 300, 140 Commonwealth Ave., Chestnut Hill, MA 02467 USA

**Keywords:** Cortical structure, Subcortical, Reliability, Fractal dimensionality, Cortical thickness, Gyrification, Structural complexity

## Abstract

Metrics of brain morphology are increasingly being used to examine inter-individual differences, making it important to evaluate the reliability of these structural measures. Here we used two open-access datasets to assess the intersession reliability of three cortical measures (thickness, gyrification, and fractal dimensionality) and two subcortical measures (volume and fractal dimensionality). Reliability was generally good, particularly with the gyrification and fractal dimensionality measures. One dataset used a sequence previously optimized for brain morphology analyses and had particularly high reliability. Examining the reliability of morphological measures is critical before the measures can be validly used to investigate inter-individual differences.

## Introduction

A growing number of studies have investigated relationships between brain morphology and inter-individual differences. An important assumption that underlies these studies is that estimates of brain morphology are reliable. While numerous studies have investigated the test–retest reliability for estimates of cortical thickness (e.g., [1–7]) and subcortical volume (e.g., [7–12]), the reliability of other measures of brain morphology has been less established and is an important topic of future research [13]. Here we measured the reliability of several measures of cortical and subcortical structures; in addition to cortical thickness and subcortical volume, we examined the reliability of estimates of cortical gyrification and fractal dimensionality.

Gyrification index is a measure of the ratio between the surface area of the cortex, relative to a simulated enclosing surface that surrounds the cortex (e.g., [14–18]). Generally, gyrification has been suggested to be an important characteristic of the human brain [15–19]. In addition to the well-known differences in cortical thickness associated with age, gyrification also differs with age [20–22]; however, age-related differences in gyrification appear to have a distinct topological distribution than thickness [20, 21]. Gyrification has also been associated with a myriad of other inter-individual measures, as reviewed by Mietchen and Gaser [14].

Structural complexity is measured as fractal dimensionality, which uses fractal geometry principles [23] to measure the complexity of brain structures (see [21]). We recently demonstrated robust age differences in the structural complexity of cortical [21] and subcortical structures [24]. Less work has been done examining the relationship between inter-individual differences and variance in complexity of cortical and subcortical regions; however, these approaches have been found to be useful in a variety of disciplines within neuroscience [25, 26].

Here we examined the test–retest reliability of several measures of brain morphology. While volumetric measures—cortical thickness and subcortical volume—have been evaluated previously, we additionally evaluated the reliability of shape-related measures, specifically gyrification and fractal dimensionality. We evaluated the FreeSurfer implementation of gyrification, as implemented by Schaer et al. [27]. This approach generates an enclosing surface around each hemisphere and computes the ‘local’ difference in surface between this surface and the pial surface of the cortex. As such, gyrification is highest over the insula and lowest over medial cortical regions. Fractal dimensionality was evaluated based on the calcFD toolbox [21], which computes fractal dimensionality using intermediate files generated as part of the standard FreeSurfer pipeline. Madan and Kensinger [21] previously compared different algorithms for calculating fractal dimensionality using simulated 3D structures, but here we instead used multiple anatomical volumes acquired from the same participant (i.e., test–retest reliability).

Structural measurements are often used to assess longitudinal changes or inter-individual differences. For instance, advancements in measuring relationships between brain morphology and inter-individual differences have become increasingly relevant as a complementary approach to fMRI, due to aging-related confounds in group comparisons [28]. More recently, age-related differences have been identified in BOLD signal variability [29, 30], which may be related to differences in cerebrovascular reactivity [31, 32]. As brain morphology research advances, it is critical to measure the reliability of these metrics using multiple volume acquisitions. For instance, if the effect of age on a morphological measure is small, poorer reliability may make the effect difficult to detect due to noise in the measure. A number of open-access databases include multiple scans on the same participants, enabling such reliability to be calculated. Appendix 1 summarizes a number of additional open-access datasets—in addition to those we consider here—that also include intersession test–retest reliability data.

Here we examined test–retest reliability from two open-access datasets in which participants were scanned several times over a short interval (i.e., intersession, intrascanner). In the first dataset, 30 participants were scanned 10 times within a 1-month period [33]. In the original work, Chen et al. sought to estimate test–retest reliability of resting-state networks across intra- and inter-individual variability of six rs-fMRI measures (CCBD [Center for Cognition and Brain Disorders] dataset). In the second dataset, 69 participants were scanned twice within a 6-month period [34]. Holmes et al. collected data for a large-scale exploration (*N* = 1570) of the relations among brain function, behavior, and genetics (GSP [Brain Genomics Superstruct Project] dataset). As one demonstration of the uses of this dataset, Holmes et al. [3] examined the relationship between cortical thickness and several measures of cognitive control.

In each of these datasets, we examined the reliability of three cortical measures: cortical thickness, gyrification, and fractal dimensionality—both of the entire cortical ribbon and across regional measures of parcellated cortex (62 regions, based on the DKT atlas; [35]). We additionally evaluated different approaches to calculating fractal dimensionality to establish the reliability of each of these approaches. Finally, reliability of volume and fractal dimensionality of segmented subcortical and ventricular structures also was evaluated. We consider each dataset separately, as would be the typical approach for examining test–retest reliability, and then discuss the conclusions reached using both datasets in the general discussion.

## Study 1: CCBD

### Procedure

#### Dataset

MR images were acquired using a GE MR750 3 T scanner at the Centre for Cognition and Brain Disorders (CCBD) at Hangzhou Normal University [33]. Thirty participants aged 20–30 years old were each scanned for 10 sessions, occurring 2–3 days apart over a 1-month period. T_1_-weighted data were acquired using a FSPGR sequence (TR: 8.06 s; TE: 3.1 ms; flip angle: 8°; voxel size: 1.0 × 1.0 × 1.0 mm). This dataset is included as part of the Consortium for Reliability and Reproducibility (CoRR; [36]) as HNU1.

#### Preprocessing of the structural data

Data were analyzed using FreeSurfer 5.3.0 (https://surfer.nmr.mgh.harvard.edu) on a machine running CentOS 6.6. FreeSurfer was used to automatically volumetrically segment and parcellate cortical and subcortical structures from the T_1_-weighted images [37–40]. FreeSurfer’s standard pipeline was used (i.e., recon-all). No manual edits were made to the surface meshes, but surfaces were visually inspected.

Cortical thickness is calculated as the distance between the white matter surface (white–gray interface) and pial surface (gray–CSF interface) [38]. Thickness estimates have previously been found to be in agreement with manual measurements from MRI images [41, 42], as well as ex vivo tissue measurements [43, 44]. Subcortical volume estimates have also been found to correspond well with manual segmentation protocols, particularly in young adults [45–52].

Gyrification was also calculated using FreeSurfer, as described in Schaer et al. [27]. Cortical regions were delineated based on the Desikan–Killiany–Tourville (DKT) atlas, also part of the standard FreeSurfer analysis pipeline [35]. Intracranial volume (ICV) was also calculated using FreeSurfer [53].

Fractal dimensionality was quantified using the calcFD toolbox (http://cmadan.github.io/calcFD/), which we previously developed and distribute freely [21, 24]. calcFD is a MATLAB toolbox that calculates the fractal dimensionality of 3D structures and was developed to work with intermediate files from the standard FreeSurfer pipeline. Apart from when otherwise stated, FD was calculated for filled structures (FD_*f*_) using the dilation algorithm. Here we additionally modified calcFD in two ways. First, we improved it to additionally calculate the fractal dimensionality of cortical parcellations for all regions delineated in the DKT atlas (see Appendix 2). An important consideration in decreasing the size of cortical parcellations, however, is that they inherently have decreased fractal dimensionality, i.e., becoming closer to a ‘truncated rectangular pyramid.’ Second, we adjusted the toolbox to calculate fractal dimensionality using the spherical harmonics (e.g., [54–58]). Additional details about this spherical harmonics approach are outlined in Appendix 3.

#### Measuring reliability

Reliability was calculated as intraclass correlation coefficient (ICC), which can be used to quantify the relationship between multiple measurements [59–62]. McGraw and Wong [63] provide a comprehensive review of the various ICC formulas and their applicability to different research questions. ICC was calculated as the one-way random effects model for the consistency of single measurements, i.e., ICC(1). As a general guideline, ICC values between .75 and 1.00 are considered ‘excellent,’ .60–.74 is ‘good,’ .40–.59 is ‘fair,’ and below .40 is ‘poor’ [64]. For the cortical parcellated regions, distributions of mean reliability measures (e.g., lower panel of Fig. 4) were compared using a Mann–Whitney *U* test, a nonparametric for testing whether two sets of values belong to the same distribution.

In the current study, we focused on regional estimates of brain morphology; a complimentary approach that we did not evaluate here is the reliability in spatial segmentation. This alternative approach evaluates the volumetric overlap between 3D structures within the same space, often quantified as a Dice coefficient (e.g., [5, 10, 48, 50, 65]). This overlap approach is often used when comparing manual and automatic segmentation protocols of the same anatomical volume; however, it can be applied to test–retest reliability by co-registering the individual anatomical volumes from the same participant to each other and comparing the resulting segmented structures’ overlap. In contrast, the present goal was to evaluate ‘summary statistics’ of the structures, such as thickness, volume, and fractal dimensionality.

### Results

#### Cortical ribbon

**Fig. 1:**
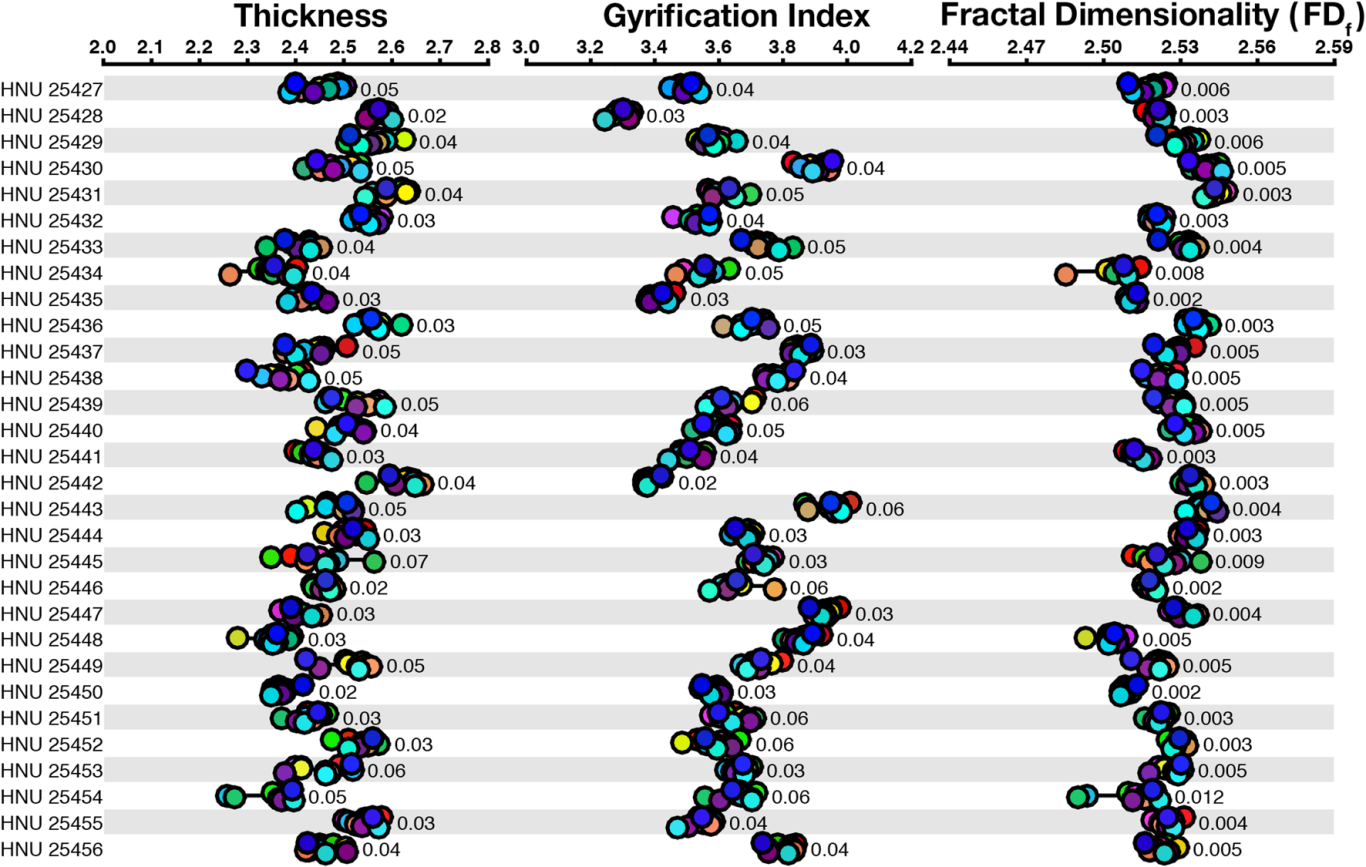
Dot plot for the structural estimates for each measure for the cortical ribbon, for the CCBD dataset. Participant labels are presented on the left, such that *each row* represents structural metrics for a single participant. Each *dot* within a measure (e.g., ‘Thickness’) represents a different scan volume. Within each row, markers in the same *color* denote measures taken from the same scan volume. Values beside each set of markers denote the mean deviation between estimates. (Color figure online)

We first examined the test–retest reliability of cortical thickness and gyrification, as shown in Fig. 1 and Table 1. Across both measures, estimates clustered closely for all scans from the same individual. This qualitative finding was corroborated by high ICC values, .816 and .945 for thickness and gyrification, respectively.

**Table 1:** Test–retest reliability (ICC) for each measure and dataset, for the cortical ribbon data

Measure	Study 1 CCBD	Study 2 GSP
Thickness (CT)	.816	.890
Gyrification (GI)	.945	.941
*Fractal dimensionality*		
Dilation filled (FD_*f*_)	.842	.936
Dilation surface	.845	.936
Boxcount filled	.799	.879
Boxcount surface	.769	.849
SPHARM surface	.977	.982

SPHARM refers to spherical harmonics. When not otherwise stated, FD_*f*_ represents FD as calculated using the dilation-filled approach

*Fractal dimensionality* We computed the reliability of five calculations of fractal dimensionality. First, we used both the dilation and box-counting algorithms, as implemented in the calcFD toolbox, for both the filled volumes and surfaces only. We additionally used a spherical harmonics (SPHARM) approach (surface only). See Appendix 3 for further details about calculating fractal dimensionality using spherical harmonics. Figure 1 shows estimates of fractal dimensionality based on the dilation-filled approach.

As shown in Table 1, we consistently found higher reliability for the dilation algorithm than the box-counting algorithm, though this difference was not statistically significant. We found higher reliability for the spherical harmonics approach; however, this approach can only be used for surfaces of structures (rather than filled volumes).

#### Cortical parcellations

**Fig. 2:**
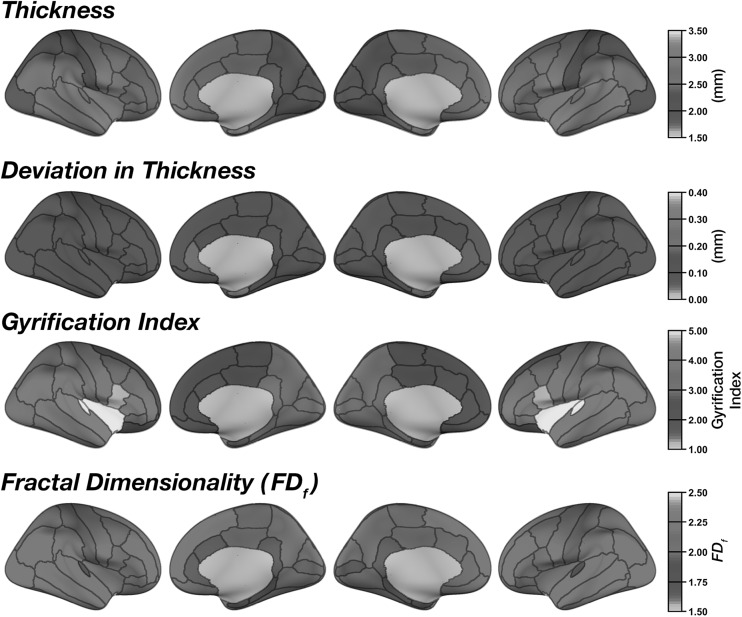
Mean regional morphology measures for each parcellated region plotted on inflated surfaces, for the CCBD dataset

**Fig. 3:**
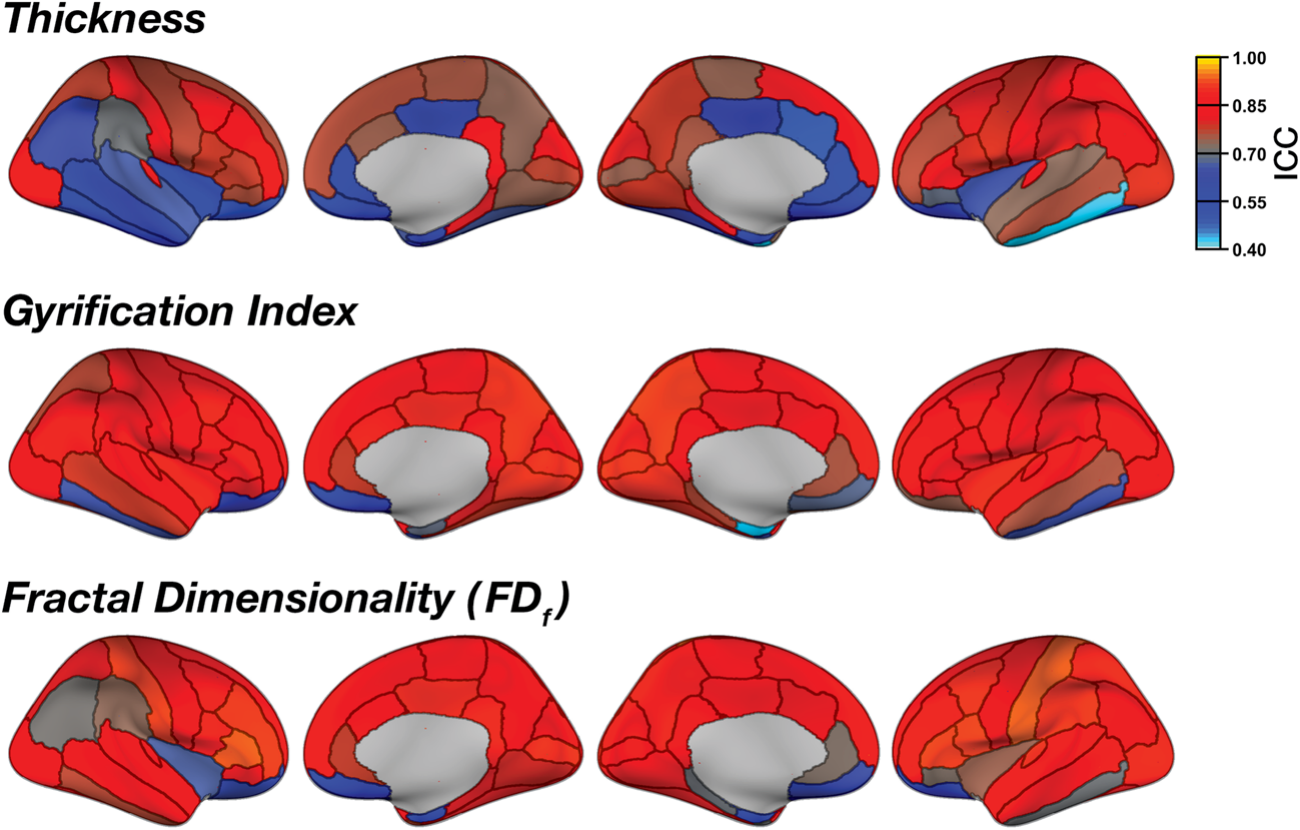
Test–retest reliability (ICC) for cortical thickness, gyrification, and fractal dimensionality of the cortical parcellations, for the CCBD dataset

**Fig. 4:**
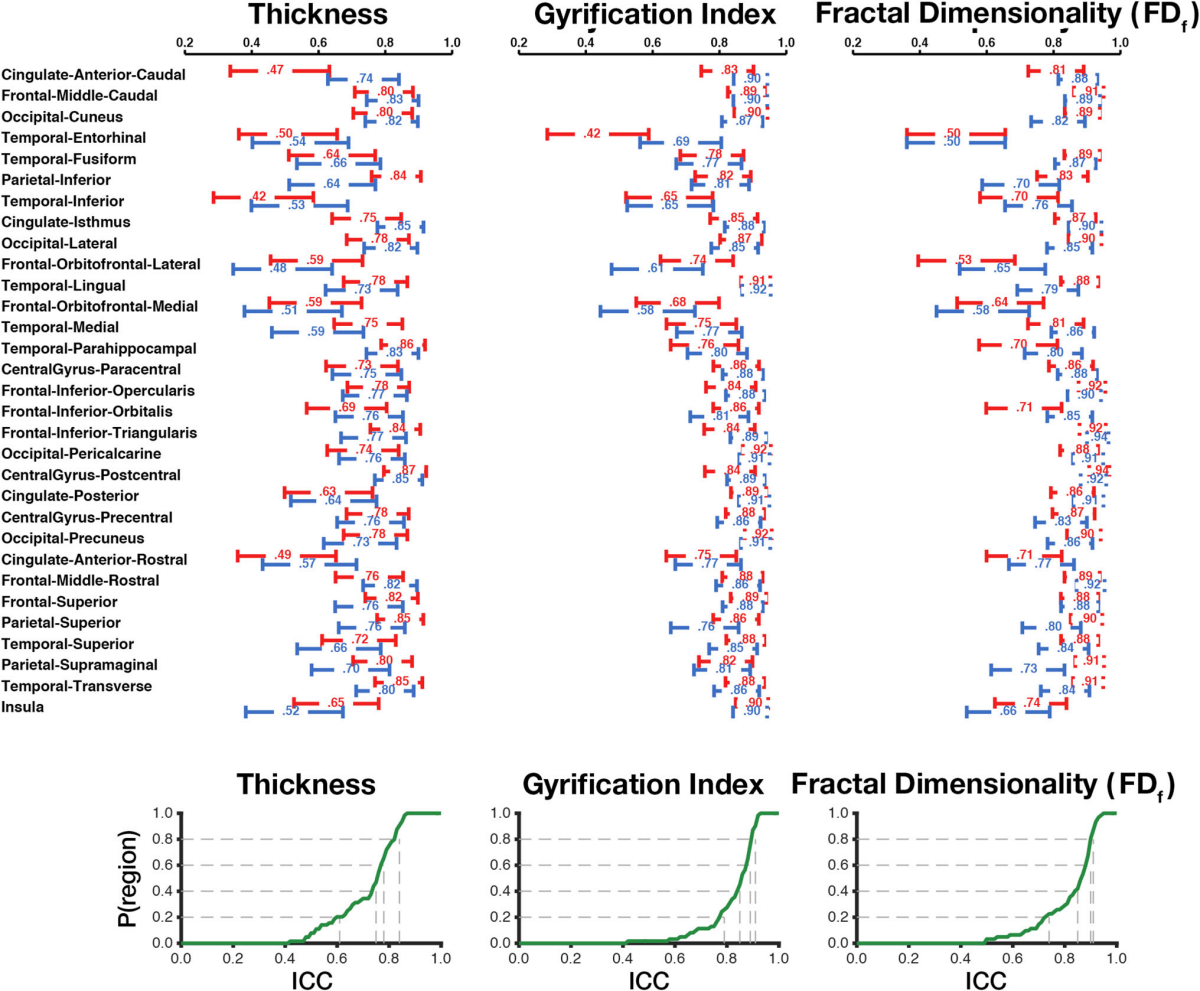
Test–retest reliability (ICC) for cortical thickness, gyrification, and fractal dimensionality of the cortical parcellations, for the CCBD dataset. *Upper* mean ICC values, with 95% confidence intervals, for each region and measure. Right hemisphere regions are displayed in *red*; left hemisphere regions are displayed in *blue*. Lower: empirical cumulative distribution functions (CDFs) of the mean ICC values. *Gray lines* show the proportion of regions with at least a mean ICC of *x*. (Color figure online)

Mean regional cortical thickness was highest in lateral temporal regions, followed by frontal regions (Fig. 2). This pattern is consistent with prior findings (e.g., [20, 21, 38, 66, 67]). Regional thickness estimates were highly consistent across regions, as shown by the low mean deviation (between scans) for each region in Fig. 2. ICC values for each region are shown in Figs. 3 and 4. Regions with the greatest intersession variability are convergent with prior reliability analyses (see [2] (Fig. 2), [3] (Fig. 1), [4] (Fig. 3), [6] (Fig. 1)). Generally, thickness estimates are less reliable around the temporal pole and would be most affected in the inferior temporal gyrus using the DKT parcellation scheme, and the anterior and medial cingulate. Thickness estimates are often highest in parietal (particularly superior parietal) and occipital cortices. Nonetheless, despite the spatial variability in thickness reliability, mean deviations are often small in magnitude, often around .10 mm (Fig. 2) (see [2] (Fig. 2)).

As expected (as in [15]), gyrification was highest in the insula and lowest over medial cortical regions (Fig. 2). Beyond this, we additionally observed greater gyrification over parietal regions, convergent with prior studies (e.g., [20, 21]). Test–retest reliability of regional gyrification was generally quite high (Figs. 3, 4) and was significantly higher for gyrification than cortical thickness [*Z* = 5.98, *p* < .001].

Regional fractal dimensionality is shown in Fig. 2. Smaller regions had lower fractal dimensionality, as smaller segmented structures inherently have less structural complexity due to both limitations MRI acquisition precision and biological constraints (also see [24]). Intraclass correlations (ICCs) are shown for each structural measure and brain region in Fig. 3; Fig. 4 shows the 95% confidence intervals of the inter-class correlations (ICCs) for each measure and region. Across regions, mean ICC was not significantly related to the size of the region for any of the measures [thickness: *r*(60) = .206, *p* = .11; gyrification: *r*(60) = .154, *p* = .23; fractal dimensionality: *r*(60) = .251, *p* = .05]. Test–retest reliability of regional fractal dimensionality was generally high (Figs. 3, 4) and was also significantly higher than for cortical thickness [*Z* = 5.46, *p* < .001]. Reliability did not differ between gyrification and fractal dimensionality [*Z* = .31, *p* = .75].

#### Subcortical structures

**Fig. 5:**
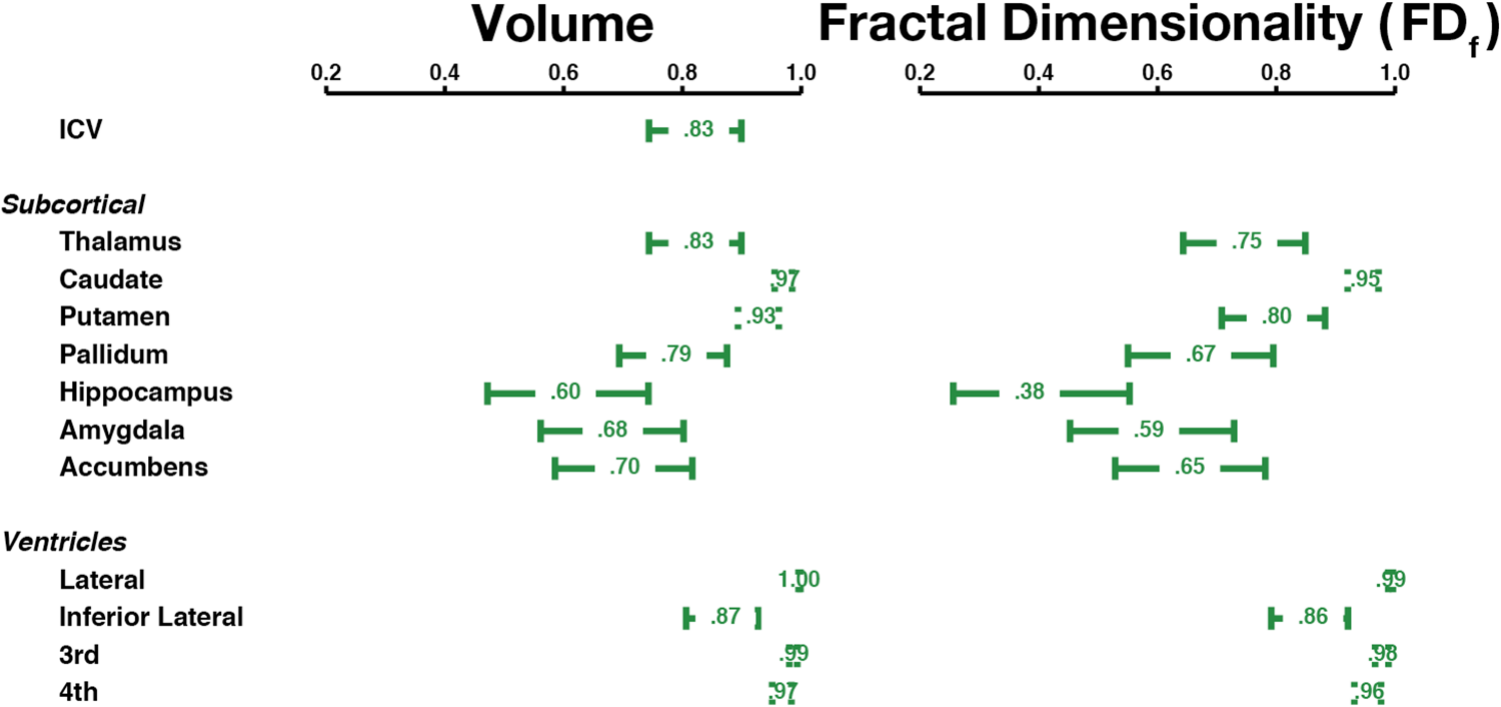
Test–retest reliability (ICC; mean and 95% confidence interval) for volume and fractal dimensionality of the subcortical structures, for the CCBD dataset

Test–retest reliability was relatively high for most structures and was quite similar for both volume and fractal dimensionality (Fig. 5). Reliability was lowest for the hippocampus; reliability was the highest for the caudate, putamen, and thalamus. Reliability estimates were significantly higher for the ventricles than the subcortical structures.

#### Summary

The results indicate that gyrification and fractal dimensionality have high test–retest reliability. Indeed, reliability using these measures was higher than for cortical thickness.

## Study 2: GSP

To further assess the replicability of these findings, we calculated these same measures in a second dataset. While this dataset had only two MRI sessions, rather than 10, this dataset used an anatomical MRI sequence that was optimized for brain morphology research (based on prior validation work assessing cortical thickness and subcortical volume) [7, 68]. While this prior validation work suggests that reliability for cortical thickness and subcortical volume should be higher for this dataset, it is not clear how these improvements to volumetric measures may influence *shape*-related measures of morphology (i.e., gyrification and fractal dimensionality).

### Procedure

#### Dataset

MR images were acquired on Siemens Trio 3 T scanners at Harvard University and Massachusetts General Hospital, as part of the Brain Genome Superstruct Project (GSP; [34]). This dataset includes 1570 participants from aged 18 to 25 years old. Test–retest reliability data were available for 69 participants who were scanned within 6 months of their first session (also see [3]). T_1_-weighted data were acquired using a MEMPRAGE sequence optimized for brain morphology (TR: 2.20 s; TE: 1.5, 3.4, 5.2, 7.0 ms; flip angle: 7°; voxel size: 1.2 × 1.2 × 1.2 mm) [7, 68].

#### Data analysis

The MR images were processed using an identical procedure as in Study 1. ICC was also evaluated using the same approach.

### Results

#### Cortical ribbon

**Fig. 6:**
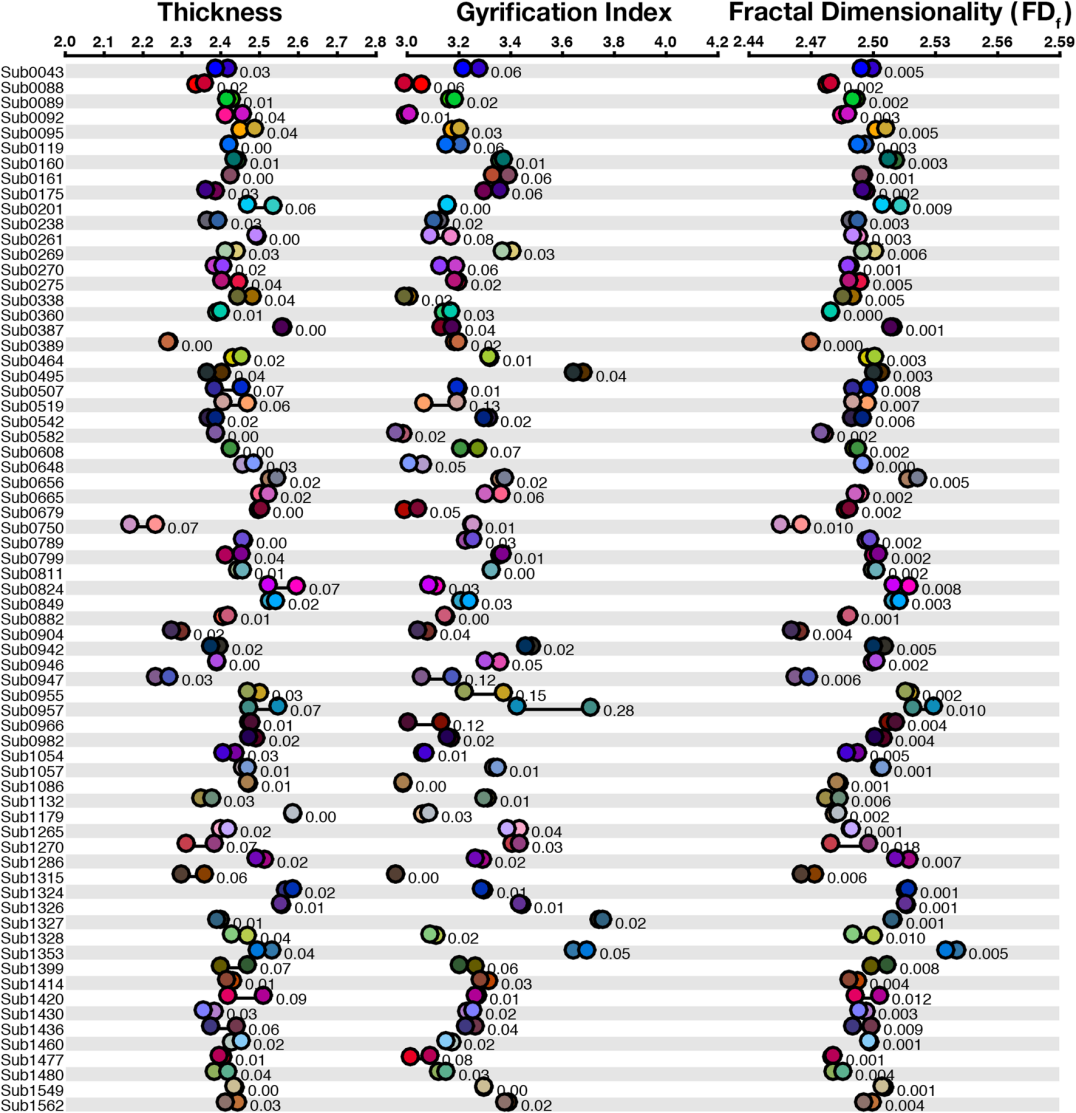
Dot plot for the structural estimates for each measure for the cortical ribbon, for the GSP dataset. *Each row* represents structural metrics for a single participant, and each dot within a measure (e.g., ‘Thickness’) represents a scan volume. Within each row, markers in the same *color* denote measures from the same scan volume, across measures. Values beside each set of markers denote the mean deviation between estimates. (Color figure online)

As shown in Fig. 6, morphology estimates from the two sessions were generally highly concordant, though estimates did markedly differ for some participants (e.g., Sub0955, Sub0957). Nonetheless, test–retest reliability (ICC) was comparable as with the CCBD dataset (see Table 1). In almost all cases, reliability was numerically higher for the GSP dataset than for the CCBD dataset, though this difference was not statistically significant.

#### Cortical parcellations

**Fig. 7:**
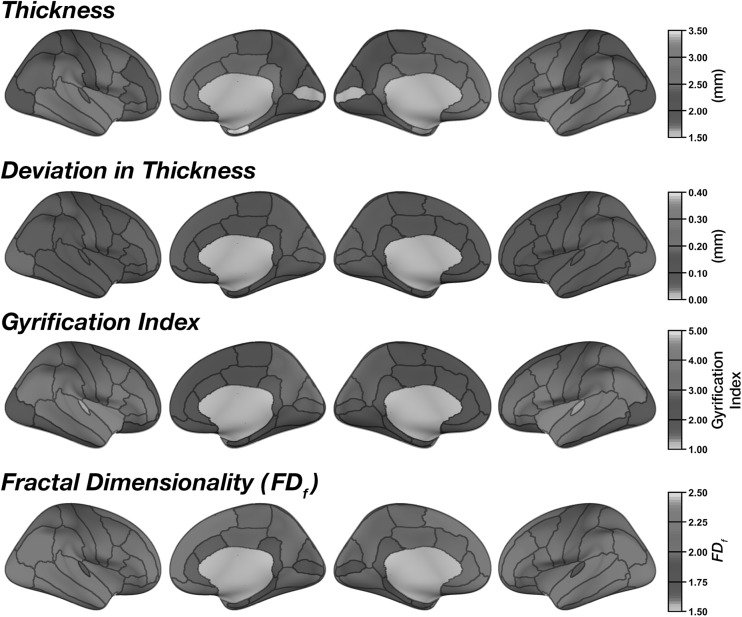
Mean regional morphology measures for each parcellated region plotted on inflated surfaces, for the GSP dataset

**Fig. 8:**
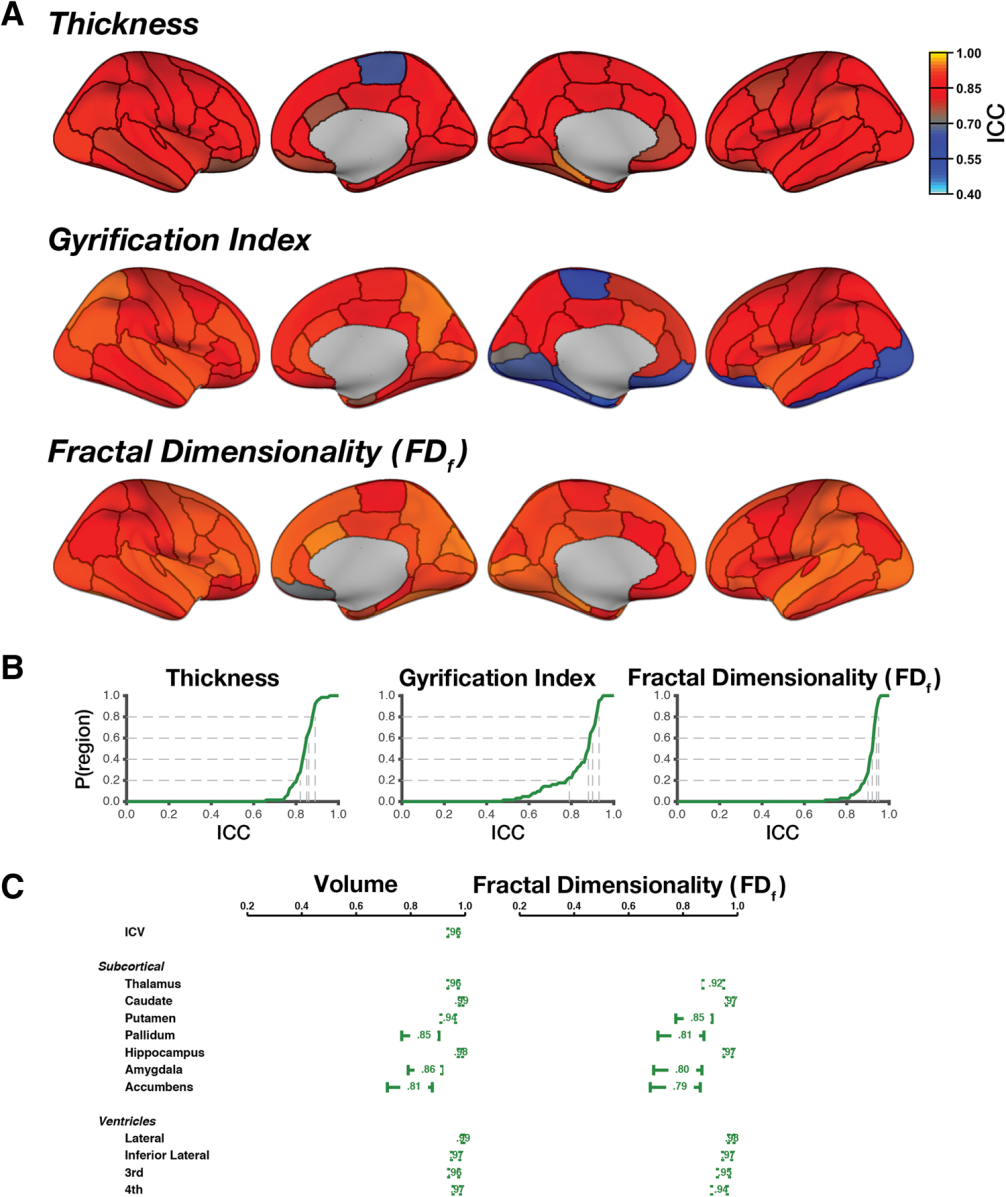
Test–retest reliability (ICC) for regional parcellations and subcortical structures, for the GSP dataset. **a** ICCs for cortical thickness, gyrification, and fractal dimensionality of the cortical parcellations. **b** Empirical cumulative distribution functions (CDFs). *Gray lines* show the proportion of regions with at least a mean ICC of *x*. (C) ICCs (mean and 95% confidence interval) for volume and fractal dimensionality of the subcortical structures

Regional estimates of thickness, gyrification, and fractal dimensionality were nearly identical between the two datasets (see Figs. 2, 7). However, it is important to note that test–retest reliability of regional estimates was very high across all regions and measures (Fig. 8a) and was indeed numerically higher than in the CCBD dataset. It is likely the increased reliability in this dataset, relative to the CCBD dataset, is related to the prior work optimizing the anatomical sequence optimized for brain morphology analyses [7, 68]. In this GSP dataset, the reliability differed between all three measures (Fig. 8b): Regional thickness had greater reliability than regional gyrification [*Z* = 2.27, *p* = .023]. Regional fractal dimensionality had greater reliability than both thickness [*Z* = 7.21, *p* < .001] and gyrification [*Z* = 4.91, *p* < .001].

#### Subcortical structures

As shown in Fig. 8c, test–retest reliability was near perfect for both volume and fractal dimensionality of the subcortical structures. The regions that had relatively lower reliability (pallidum, amygdala, accumbens) were also relatively lower in Study 1, demonstrating the replicability of lower test–retest reliability in these regions—at least when segmented using FreeSurfer’s automated algorithms. Reliability was particularly high for the hippocampus and was significantly higher than in the CCBD dataset (Study 1).

## Discussion

Here we evaluated the test–retest reliability of several brain morphology measures using open-access datasets. Prior work had examined the reliability of volumetric measures—cortical thickness and subcortical volume; however, the present study is the first to assess reliability of *shape*-related measures, gyrification and fractal dimensionality.

Both datasets showed relatively high reliability for all morphology measures and additionally revealed that reliability was particularly good for the gyrification and fractal dimensionality measures. Additionally, we provide empirical evidence that the dilation approach for calculating fractal dimensionality was superior in reliability to the ‘standard’ box-counting method. These findings held across two datasets, but reliability was particularly good in the GSP dataset, where the anatomical sequence had been previously optimized for use in brain morphology studies.

Although reliability was good in these datasets, there is still the question of how reliability may be increased in future studies. A number of factors have been found to influence estimates of brain morphology. Broadly, these factors can be divided into three categories: MR acquisition, biological, and analysis related. For MR acquisition, there are not yet enough datasets available to systematically examine how reliability is affected by the particular acquisition protocols, although the current data suggest that sequences previously optimized for brain morphology analyses (i.e., those used in GSP dataset) will have better reliability. Another acquisition-related factor is head movement; movement has been shown to lead to decreased estimates of cortical thickness [69–72], though it is unclear how movement would affect measures of gyrification and fractal dimensionality. This issue may become less critical in future studies, as recent advances in structural imaging have been able to attenuate movement-related artifacts (e.g., [73–76]). Morphological measures can also be influenced by biological confounds, such as hydration [77–80] or circadian rhythms [81, 82]. Additionally, it is important to control for variations in analysis software and operating system, which can also affect brain morphology estimates [65, 83, 84].

While the surface reconstructions were visually inspected, the surfaces were not manually edited, for two reasons. First and foremost, the quality of the automatic reconstructions was judged to be acceptable and did not require manual intervention. While manual editing is more necessary with older adult and patient populations, all of the individuals included in the present work were young adults. Additionally, manual editing introduces a subjective component and is often not conducted in studies of reconstruction reliability [2, 5, 6, 46], though some reliability studies have included minimal manual editing [4, 7]. Given that no manual editing was conducted, the reliability estimates presented here may serve as a lower bound, where manual editing would be expected to increase reliability [4, 6]; however, there is evidence that editing may not sufficiently influence regional estimates [85, 86].

Fractal dimensionality was used here as a measure of the complexity in the shape of a structure. Results indicate that this measure was generally more reliable than volumetric morphological measures, likely because fractal dimensionality is influenced by both shape and volumetric characteristics that often covary [21, 24, 87–89]. By pooling from both of these characteristics, fractal dimensionality appears to be more reliable and should be considered in future research investigating the relationship between brain morphology and inter-individual differences.

In sum, here we evaluated the reliability of several brain morphology estimates using two open-access datasets. Reliability was generally high, providing support for using gyrification and fractal dimensionality measures to evaluate inter-individual or between-sample differences in morphology.

## Figures and Tables

**Fig. 9:**
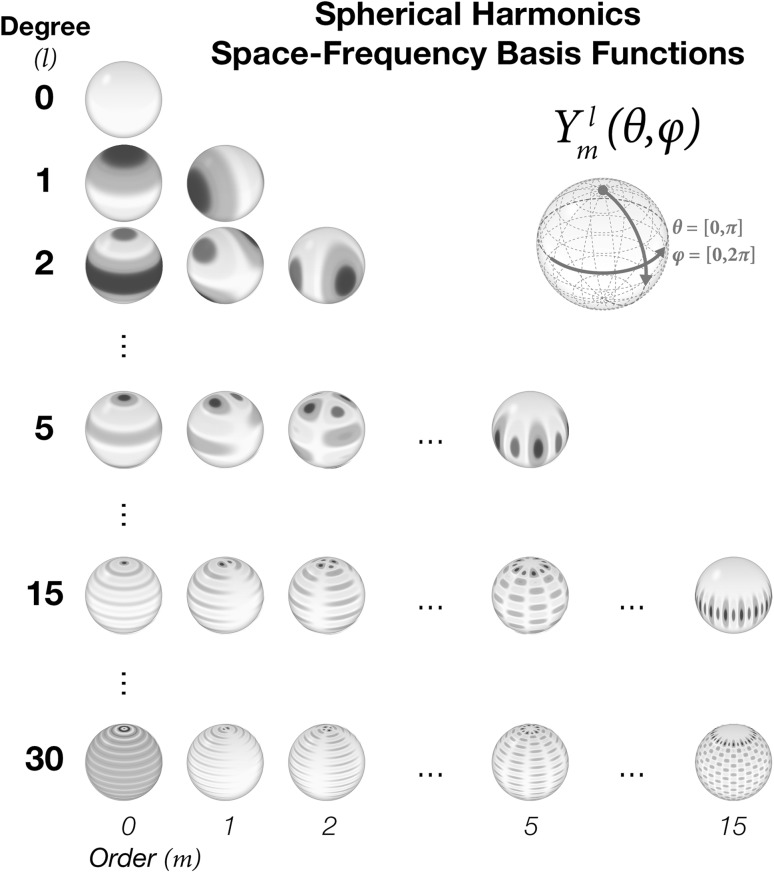
Spherical harmonics space–frequency basis functions, organized by degree (*l*) and order (*m*)

**Fig. 10:**
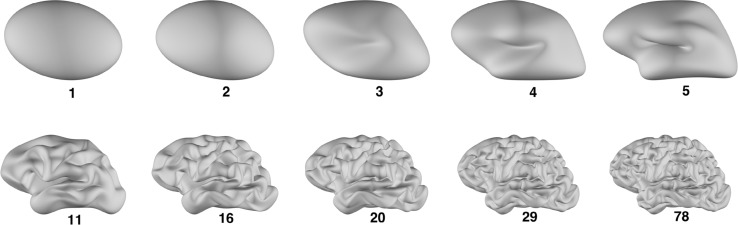
Reconstructed cortical surface for the left hemisphere for one participant, with the maximum number of degrees as denoted under each surface

## Appendix 1

See Table 2.

**Table 2:** List of open-access datasets that include intersession test–retest structural MRIs

References	*N*	Notes
*Analyzed TRT datasets*		
Chen et al. [33]	30	Ten sessions acquired over 1-month period (2–3 days between sessions); part of CoRR (HNU1; see below)
Holmes et al. [34]	69	Two sessions within 6-month period; part of larger dataset with *N* = 1570; also see Holmes et al. [3]
*Additional TRT datasets*		
Boekel et al. [99]	34	Two sessions within same day; subsample of *N* = 15 had a third session in same day and a 2-week follow-up
Marcus et al. [100]	20	Three–four volumes within session, for two sessions within a 90-day period; part of larger cross-sectional dataset of aging and dementia with *N* = 416
Morey et al. [11]	23	Two sessions within single day, follow-up in 7–9 days with another two sessions within single day
Gorgolewski et al. [101]	22	Two sessions 1 week apart, three rs-fMRI scans per session (includes high-res prefrontal cortex scan); acquired on a 7 T scanner
Landman et al. [102]	21	Two sessions within single day; multiple sequences
Gorgolewski et al. [103]	10	Two sessions acquired 2–3 days apart
*Highly sampled individual participant datasets*		
Maclaren et al. [104]	3	Two volumes within single session, for each of 20 sessions over 1-month period
Poldrack et al. [105]	1	One hundred and four sessions; scanned intermittently over 18 months (10 usable T_1_ volumes); also see Laumann et al. [106]
Choe et al. [107]	1	One hundred and fifty-eight sessions; scanned weekly for 3.5 years
Froeling et al. [108]	1	Eighteen sessions, comprising 8000 dMRI volumes (5 sessions included T_1_ volumes)
*Datasets part of CoRR*		
Zuo et al. [36]	–	Consortium for Reliability and Reproducibility (CoRR), aggregates many TRT datasets
Orban et al. [109]	80	Two volumes within single session, for each of two sessions within 3-month period; part of CoRR (UM1)
Lin et al. [110]	57	Two sessions within 6-week period; part of CoRR (BNU1)
Huang et al. [111]	61	Two sessions within 6-month period; part of CoRR (BNU2)

Note, nearly all of these datasets also include test–retest rs-fMRI data, some additionally collected task-based fMRI data

## Appendix 2

### Generating DKT volumes for the calcFD toolbox

calcFD toolbox (build 28 [and above]; http://cmadan.github.io/calcFD/) can calculate the fractal dimensionality for parcellated cortical regions based on the DKT atlas. This is done based on the aparc.DKTaltas40+aseg.mgz volume, which must first be generated using FreeSurfer, but is not part of the standard analysis pipeline. The FreeSurfer command to accomplish this is:

$$\texttt{mri\_aparc2aseg}-\mathtt{s [SUBJECTID]}-\texttt{annot aparc.DKTatlas40}$$

where [SUBJECTID] corresponds to the individual subject folder.

After these volumes have been generated by FreeSurfer, the calcFD toolbox only needs the options to be set to DKT (set options.aparc to ‘DKT’).

## Appendix 3

### Measuring fractal dimensionality via spherical harmonics

In addition to the fractal dimensionality measures considered in Madan and Kensinger [21], we additionally considered an approach based on spherical harmonics (often abbreviated as ‘SPHARM’). Yotter et al. [58] demonstrated that fractal dimensionality can be calculated using spherical harmonics and compared this to the standard box-counting approach. Importantly, they found that the spherical harmonics approach was more robust to rotations of the structure than the box-counting method. We additionally implemented this approach when evaluating the test–retest reliability of fractal dimensionality estimates.

Briefly, spherical harmonics can be used to reconstruct complex 3D surfaces based on space–frequency deformations to a sphere, based on similar principles as used to reconstruct complex wave functions using Fourier transforms. Some of the spherical harmonics basis functions are shown in Fig. 9. Chung [54, 90] provides a comprehensive introduction to these principles.

We used weighted spherical harmonics, a generalized form of traditional spherical harmonics, which substantially reduces ringing artifacts related to the Gibbs phenomenon [54, 56]. Previous studies have used spherical harmonics to study the shape of cortical and subcortical structures (e.g., [55–57, 87, 89, 91–98]), but spherical harmonics have not been connected with fractal dimensionality approaches until recently [58].

The fractal dimensionality approach we took, using spherical harmonics, was conducted based on weighted spherical harmonics equations provided by Chung et al. [54–56] and the fractal dimensionality equations from Yotter et al. [58]. The spherical parameterization provided by FreeSurfer (?h.sphere, ?h.pial) is used as the input surfaces for this processes. Cortical surfaces were reconstructed for each hemisphere with a maximum degree of *l* = {11, 16, 20, 29} (a subset of those suggested by [58]) and a bandwidth of *σ* = .001 (as recommended by [54]). The reconstruction of one hemisphere, across a variety of degrees, is shown in Fig. 10. The calculations involved in reconstructing cortical surfaces using weighted spherical harmonics are discussed in detail in Chung [54].
